# Enhancement of the Mechanical Properties of Polyimide Film by Microwave Irradiation

**DOI:** 10.3390/polym11030477

**Published:** 2019-03-12

**Authors:** Ju-Young Choi, Seung-Won Jin, Dong-Min Kim, In-Ho Song, Kyeong-Nam Nam, Hyeong-Joo Park, Chan-Moon Chung

**Affiliations:** Department of Chemistry, Yonsei University, Wonju, Gangwon-do 26493, Korea; cjy0510@yonsei.ac.kr (J.-Y.C.); jinsw0906@yonsei.ac.kr (S.-W.J.); dmkimr@yonsei.ac.kr (D.-M.K.); segunda@yonsei.ac.kr (I.-H.S.); nkn001@yonsei.ac.kr (K.-N.N.); hyeongjoo1016@yonsei.ac.kr (H.-J.P.)

**Keywords:** polyimide film, microwave, molecular weight, tensile strength, film flexibility, end group reaction

## Abstract

Polyimide films have conventionally been prepared by thermal imidization of poly(amic acid)s (PAAs). Here we report that the improvement of tensile strength while increasing (or maintaining) film flexibility of polyimide films was accomplished by simple microwave (MW) irradiation of the PAAs. This improvement in mechanical properties can be attributed to the increase in molecular weight of the polyimides by MW irradiation. Our results show that the mechanical properties of polyimide films can be improved by MW irradiation, which is a green approach that requires relatively low MW power, very short irradiation time, and no incorporation of any additional inorganic substance.

## 1. Introduction

Polyimide films have been widely used as high-performance polymer materials in flexible printed circuit boards, heat-releasing sheets, and organic light-emitting diode displays due to their excellent heat resistance, chemical resistance, electric characteristics, and mechanical strength [1,2,3]. There has recently been an intense effort to improve the mechanical properties of the polyimide films to enhance their applicability in various industries. Most studies have focused on the reinforcement of the polyimide films with inorganic substances such as graphene [4,5], graphene oxides [6,7,8], clays [9,10], silica [11,12,13], and carbon nanotubes [14]. In the majority of cases, by the incorporation of stiff inorganic substances, the tensile strength and modulus were increased, and the elongation at break was reduced. A few studies reported increased strength, modulus, and elongation at break values of the organoclay-reinforced polyimide films [15,16]. Because the modulus and elongation at break properties represent film flexibility [17,18,19,20], reinforcement with inorganic materials generally results in the reduction of film flexibility. Therefore, in fields where film flexibility is important, improvement of the tensile strength while maintaining or increasing film flexibility is desirable. Furthermore, mechanical-property improvement achieved without the addition of any inorganic material is more advantageous. No use of additional inorganic substances leads to a simpler film forming process, less energy consumption, and less use of auxiliary chemicals, and thus, it is considered to be a green approach [21].

Microwave (MW) energy has been used for many years in chemistry, primarily because MW irradiation leads to faster reaction rates compared to conventional heating systems. In addition, the direct heating of molecules under MW irradiation leads to uniform heating, resulting in reduced side reactions, cleaner products, and higher yields [22,23,24,25,26,27]. The MW-assisted green approach is also being used in polymer research and industry [23,28,29,30,31,32]. MW-mediated polymerization, which involves the self-heating of monomers or reaction medium by MW irradiation, has been shown to result in improved polymerization speeds and low-temperature polymer curing. In addition, reaction control and synthetic yield have both been improved by many studies.

Polyimide films are usually prepared using a conventional two-step synthesis method in which tetracarboxylic dianhydride and diamine are polymerized in an organic solvent to produce a poly(amic acid) (PAA) solution. The PAA solution is drop-cast onto a substrate and then imidized into polyimide film by thermal treatment. Since the anhydride, amino, and carboxyl groups in the monomers and PAAs exhibit polarity, they are sensitive to MW energy. Therefore, efforts have focused on preparing polyimides and polyimide composites using MW equipment [32,33,34,35,36,37,38,39,40,41]. However, there has been no study of the potential improvements of the mechanical properties of polyimides using MW energy.

In this study, we found that the mechanical properties of polyimide films were enhanced by MW irradiation. The advantages of this method include the simplicity of MW irradiation for a short time and no incorporation of inorganic substances into the polyimide films. We investigated the viscosities and the mechanical and thermal properties of MW-irradiated polyimide films to find the optimum conditions for MW treatment. Model studies were performed to explain the reason for the MW-assisted improvement of mechanical properties of the polyimide films.

## 2. Materials and Methods 

### 2.1. Materials and Instruments

Pyromellitic dianhydride (PMDA), 3,3′,4,4′-biphenyltetracarboxylic dianhydride (BPDA), benzophenone-3,3′,4,4′-tetracarboxylic dianhydride (BTDA), 4,4′-(hexafluoroisopropylidene)diphthalic anhydride (6FDA), and 4,4′-oxydianiline (ODA) were purchased from Merck (Seoul, Korea) and were used as received. Additionally, 1,2,4,5-cyclohexanetetracarboxylic dianhydride (HPMDA) was purchased from Samsung Lab (Seoul, Korea) and 1-methyl-2-pyrrolidone (NMP) (Daejung Chemicals & Metals, Gyeonggi-do, Korea) was distilled in reduced pressure and kept under nitrogen until use. A Magic Chef MW oven (MEM-25S, GKA International Inc., Gyeonggi-do, Korea) was used for MW irradiation. The oven operated at a frequency of 2.45 GHz with a fixed power output of 80, 240, 400, or 640 W. Thermal treatments were conducted with a vacuum oven (Jeio Tech OV-01, Jeio Tech Co., Ltd., Seoul, Korea).

### 2.2. Characterization

Proton nuclear magnetic resonance (^1^H NMR) analysis was performed using DMSO-*d*_6_ and a Bruker Avance II 400 MHz spectrometer (Bruker Corporation, Billerica, MA, USA). Elemental analysis was conducted with an EA 1108 CHNS-O (Fisons Instruments, Inc., Ipswich, UK). Fourier transform infrared (FT-IR) spectra were acquired using a PerkinElmer Spectrum One B FT-IR spectrometer (PerkinElmer, Inc., Waltham, MA, USA). Thermal analyses were carried out under nitrogen using a Shimadzu TGA-50 instrument (Shimadzu Corporation, Kyoto, Japan) at a heating rate of 10 °C/min. An infrared thermometer (35639-20, OAKTON Instruments, Vernon Hills, IL, USA) was used to measure the temperature of MW-irradiated samples. Inherent viscosities were determined at 30 °C using a Cannon-Fenske viscometer (Cannon Instrument Company, State College, PA, USA) and samples of concentration 0.50 g/dL in concentrated sulfuric acid were used. An average of three individual determinations was used for each sample. A micrometer (293-348 IP65, Mitutoyo Corporation, Kawasaki, Japan) was used to measure film thickness. The tensile properties of the films were determined using a universal testing machine (UTM) (QC-505M1, Daeha Trading Co., Ltd., Seoul, Korea). A strain rate of 2 cm/min and a 3 cm gauge were used. The measurements were performed using 0.5 cm wide, 6 cm long, and ca. 0.08 mm thick films at room temperature. An average of five individual measurements was used for each sample. Field emission scanning electron microscopy (FE-SEM) was carried out using a SU-70 (Hitachi, Ltd., Tokyo, Japan) with an acceleration voltage of 30 kV and a working distance in the range of 11.1–11.5 mm. The samples were sputter-coated with platinum. 

### 2.3. Preparation of PAAs

PMDA (2.181 g, 0.0100 mol) and ODA (2.002 g, 0.0100 mol) in NMP (36.6 mL) were charged into a dried 100 mL three-neck round-bottom flask under nitrogen. The resulting solution was stirred at 0 °C for 1 h and then further stirred at room temperature for 23 h, yielding a clear viscous solution of PAA-PO. The PAA-PO solution was used to prepare PO polyimide films or powders (see below). The other PAA solutions were prepared in a similar manner using different combinations of dianhydrides and ODA.

### 2.4. Preparation of Polyimide Films

The PAA-PO solution obtained above was drop-cast onto slide glasses and then irradiated with MW. MW irradiation was carried out at 80, 240, 400, or 640 W for 1–120 min. Next, thermal imidization was performed by stepwise heating of the PAA solutions at 50, 100, and 150 °C. The samples were kept at each temperature for 60 min. The resultant films were finally heated at 250 °C for 2 h to obtain PO polyimide films. The imidized films were cooled to room temperature and put in water for 1 h to make them easy to peel off. The resulting films were dried at 100 °C for 1 h in a vacuum oven. To prepare the Control-PO films, the PAA-PO solution obtained above was drop-cast onto slide glasses and then thermally imidized without MW irradiation. The thermal imidization procedure was the same as that described above. The other polyimide films based on different combinations of dianhydrides and ODA were synthesized in a similar fashion. The thicknesses of the polyimide films were measured to be about 0.08 mm.

### 2.5. Preparation of PAA or Polyimide Powders

The PAA-PO solution obtained above was drop-cast onto slide glasses and then irradiated with MW. MW irradiation was carried out at 80, 240, or 400 W for 1–120 min. The PAA-PO solution (irradiated or non-irradiated with MW) was poured into distilled water, forming a precipitate that was collected by filtration. The precipitate was washed with water (100 mL) and methanol (100 mL) and then dried in a vacuum, yielding PAA-PO in powder form. The other PAA powders were synthesized in a similar fashion using different combinations of dianhydrides and ODA. Thermal imidization was performed by stepwise heating of the PAA powders in a vacuum oven. The temperature was increased stepwise to 50, 100, and 150 °C. The samples were allowed to stand at each temperature for 1 h. Imidization was completed by heating at 250 °C for 2 h, yielding the polyimide powders. The prepared PAA and polyimide powders were used in FT-IR spectroscopy.

### 2.6. Determination of Degree of Imidization

The degree of imidization (DI) was determined by FT-IR spectroscopy using PAA and polyimide powders. The DI was calculated using Equation (1) [42,43,44].

(1) $$\mathrm{DI}\;\left(\%\right)=\left[(A_{1377}/A_{1500})/{\left(A_{1377}/A_{1500}\right)}_{300\;{}^{\circ}C}\right]\times 100,$$

where A_1377_ and A_1500_ are the absorbance values of the 1377 and 1500 cm^−1^ absorption bands of PAA-PO or PO, respectively. The 1377 cm^−1^ band is attributed to imide C–N stretching, and the 1500 cm^−1^ absorption band (due to aromatic C=C stretching) is used as an internal standard to normalize the variations. The band absorbance ratio of a PO polyimide prepared by thermal imidization (100 °C/1 h, 200 °C/1 h, and 300 °C/2 h) of PAA-PO was considered to be equivalent to that of the completely imidized polyimide, and therefore, it was used as a reference ratio value [42,43,44,45,46]. In order to prepare the completely imidized reference sample, the imidization temperature for the reference sample (up to 300 °C) was higher than that for the test samples (up to 250 °C).

### 2.7. Statistical Analysis

The mechanical property data were analyzed using a R Studio (version 1.1.453, RStudio, Inc., Boston, MA, USA) software. The data of PO polyimides were analyzed by means of one-way ANOVA followed by Tukey’s honestly significant difference (HSD) test. The other mechanical data were analyzed by means of an independent-samples *t* test. *P*-values less than 0.05 were considered statistically significant.

## 3. Results and Discussion

### 3.1. Preparation and Characterization of Polyimides

**Scheme 1:**
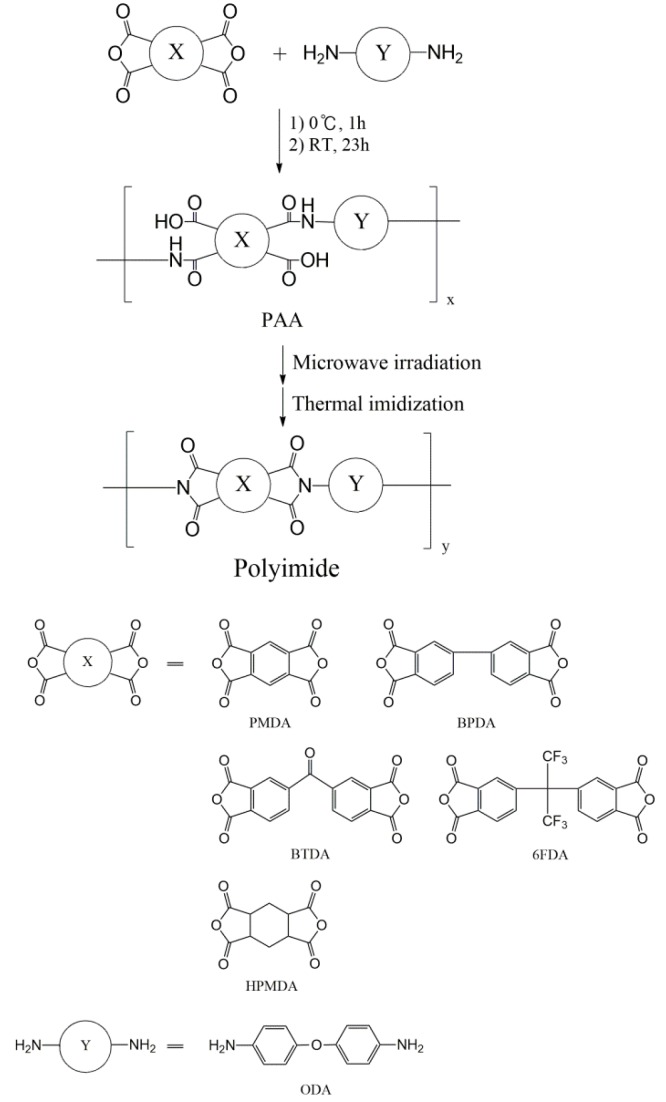
Synthesis of polyimides.

**Figure 1:**
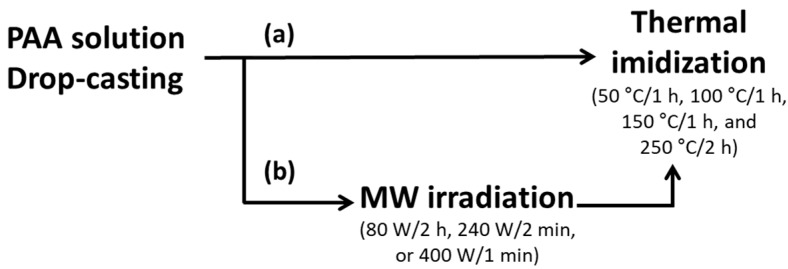
Schematic diagram of the preparation of polyimide films: (**a**) Control polyimides and (**b**) MW-treated polyimides.

The preparation procedure of polyimide films is illustrated in Scheme 1 and Figure 1. A poly(amic acid) of PMDA/ODA (PAA-PO) was first prepared by polymerization of the two monomers at a molar ratio of 1:1 in NMP. The resulting PAA-PO solution was drop-cast onto slide glasses, and MW irradiation was performed. When the PAA-PO solution within the flask or vial was irradiated, precipitation was very likely to occur. The MW irradiation of the drop-casted solution was performed in a commercial domestic MW oven. Domestic MW ovens have often been used in MW-assisted polymerizations [29,31,47]. Subsequent thermal treatment of the MW-treated PAA-PO solution yielded PO polyimide films. The other polyimide (BPO, BTO, 6FO, and HPO) films were prepared in a similar fashion from different combinations of dianhydrides and ODA (BPDA/ODA, BTDA/ODA, 6FDA/ODA, and HPMDA/ODA, respectively). For comparison, control polyimides were prepared using the conventional two-step synthesis method without MW irradiation (Figure 1). PAA and polyimide powders were also prepared for the characterization of their chemical structure by FT-IR spectroscopy (see below). Reliable gel permeation chromatography (GPC) data could not be obtained for the MW-treated PAA samples, and therefore, inherent viscosity of the MW-treated polyimides was measured in concentrated sulfuric acid (see below).

**Figure 2:**
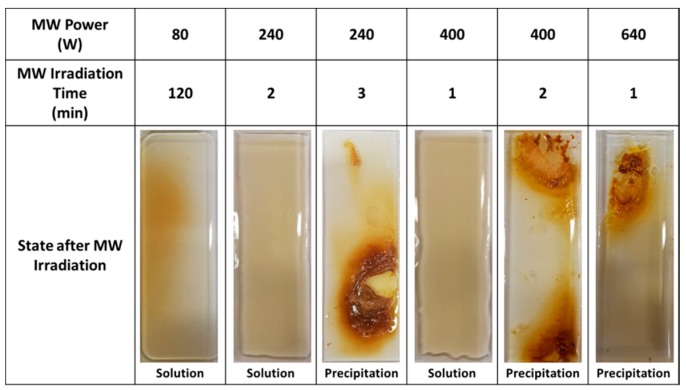
States of the PAA-PO solutions after MW irradiation. The MW irradiation was performed with drop-casted PAA-PO solutions.

We optimized the MW irradiation conditions by varying the MW power or irradiation time (Figure 2). The MW oven operated at fixed power output of 80, 240, 400, or 640 W. Upon irradiation of the PAA-PO solutions at 240 W for 3 min, at 400 W for 2 min, or at 640 W for 1 min, precipitation was observed in the PAA-PO solutions because of the high energy and/or longer irradiation time. Further irradiation was not performed because uniform films could not be obtained from these precipitate-containing mixtures. However, the PAA-PO solutions remained homogeneous after irradiation at 80 W for 120 min, at 240 W for 2 min, or at 400 W for 1 min (further irradiation at 80 W was not conducted because it required a very long irradiation time). Uniform films could be prepared when the PAA-PO solutions did not undergo precipitation after MW irradiation. Therefore, we conducted the following experiments using the MW irradiation conditions in which the PAA-PO solutions remained homogeneous. 

PO films were prepared by MW irradiation of a drop-casted PAA-PO solution, followed by thermal imidization, and the MW irradiation conditions are shown in Table 1. A control PO (Control-PO) film was also prepared from PMDA and ODA using the conventional two-step method without MW irradiation. Because the POs were insoluble in organic solvents, the inherent viscosities of PO films were measured using concentrated sulfuric acid. The viscosity measurement enables indirect comparison of the molecular weights of the POs. The viscosity of PO-80-120m (1.64 dL/g) and PO-240-2m (1.68 dL/g) was much higher than that of the Control-PO (1.23 dL/g) (Table 1). PO-400-1m exhibited slightly higher viscosity than the Control-PO. These results imply that the molecular weights of the polyimides were increased by MW irradiation.

**Figure 3:**
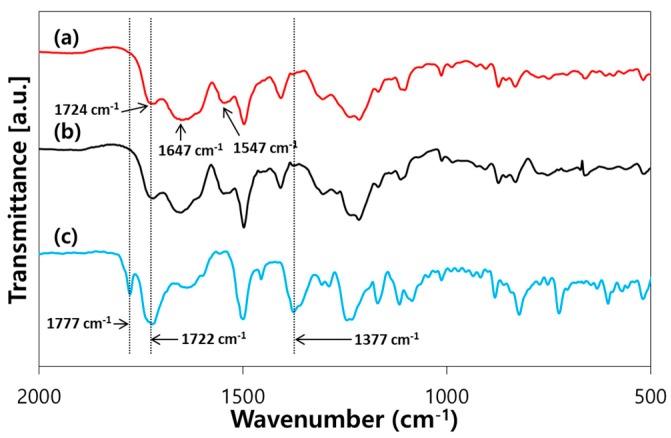
FT-IR spectra of (**a**) PAA-PO powder, (**b**) PAA-PO-240-2m powder, and (**c**) PO-240-2m powder.

The chemical structures of PAA-PO and PO were confirmed by FT-IR spectroscopy and elemental analysis (EA) (Figure 3 and Table S1). The FT-IR spectrum of PAA-PO showed absorption bands at 1724 cm^−1^ owing to C=O stretching (carboxyl), 1647 cm^−1^ owing to C=O stretching (amide), and 1547 cm^−1^ owing to C–N stretching (amide). This suggests the formation of PAA (Figure 3a). PO-240-2m, the thermal imidization product of PAA-PO-240-2m, exhibited characteristic imide absorption bands at 1777 cm^−1^ owing to imide C=O asymmetric stretching, 1722 cm^−1^ owing to imide C=O symmetric stretching, and 1377 cm^−1^ owing to imide C–N stretching, indicating the formation of polyimide (Figure 3c). FT-IR spectra of the other polyimides are shown in Figure S1. The EA values of the control polyimides and the MW-treated polyimides agreed well with the calculated values for the corresponding structures (Table S1). On the other hand, PAA-PO-240-2m, which was obtained from PAA-PO by MW irradiation at 240 W for 2 min, showed absorption bands similar to those of PAA-PO (Figure 3b). However, PAA-PO-240-2m also exhibited a small band at 1377 cm^−1^. Since this compound was formed by MW irradiation, this result suggests that partial imidization of the PAA occurred during MW irradiation.

As mentioned above, the imidization of PAAs was shown to occur to some extent during the MW irradiation [48]. The degrees of imidization (DIs) of the PAA-POs were analyzed by FT-IR spectroscopy (Figures S2 and S3). Table 2 shows the DIs of PAA-POs irradiated under various conditions. The DIs of PAA-PO-400-1m and PAA-PO-240-2m were 6% and 10%, respectively. When the samples were irradiated with 80 W of energy for 120 min, the DI reached 11% due to the longer irradiation time. Despite the partial imidization, the PAA solutions remained homogeneous, and no precipitation was observed. The DIs of the precipitated PAA-POs was also analyzed by FT-IR spectroscopy (Figure S3 and Table 2). PAA-PO-240-3m, PAA-PO-400-2m, and PAA-PO-640-1m showed DI values of 50%, 14%, and 20%, respectively, which are higher than those of the soluble PAA-POs. It is considered that the precipitation of the PAA-POs can be (partly) attributed to the increase in their DIs due to the prolonged MW irradiation and/or higher MW power.

DIs were also measured for the polyimide powders prepared by thermal imidization of the MW-irradiated PAAs: All the polyimides revealed almost 100% DIs. This indicates that the polyimide films prepared under the same imidization conditions were fully imidized.

### 3.2. Properties of Polyimides

The mechanical properties of the PO films were investigated next by determining their stress–strain curves (Figure S4) with the use of a universal testing machine (UTM). The average values of five different measurements for each polyimide are listed along with standard deviations in Table 3. PO-80-120m and PO-240-2m exhibited higher tensile strength than Control-PO and PO-400-1m (*p* < 0.05), and PO-240-2m showed the highest strength (*p* < 0.05). Elongation at break values of PO-240-2m and PO-80-120m were approximately equal (*p* > 0.05), and the values were higher than those of Control-PO and PO-400-1m (*p* < 0.05). The PO-240-2m film showed a 22% higher tensile strength and a 32% higher elongation at break than Control-PO film. It should be noted that great improvement in both tensile strength and elongation at break of the PO film was achieved. Furthermore, the improvement was possible by MW irradiation with relatively low power (240 W) for a very short time (2 min). The enhanced mechanical properties can be explained by the increase in polyimide molecular weight by MW irradiation (Table 1). In general, the mechanical strength of a polymer improves as its molecular weight increases because intermolecular force and chain entanglement density increase with increasing molecular weight [49,50,51,52]. Moreover, the increase in entanglement density of the polyimide chains is understood to confer increased elongation at break, i.e., increased film flexibility [17,18,53,54].

On the other hand, there was no significant difference between tensile modulus values of the MW-treated PO films and Control-PO film (*p* > 0.05) (Table 3). This indicates that the modulus property of the MW-treated PO films was maintained with increasing molecular weight. A similar tendency for the tensile modulus of a polymer (poly(3-hexylthiophene)) film to saturate with increasing molecular weight has been reported using a molecular dynamics simulation [55]. It has also been shown that the tensile modulus of a poly(3-hexylthiophene) film can be maintained or decrease with increasing molecular weight above an entanglement chain length [56].

Thermogravimetric analysis (TGA) was conducted to investigate the effect of the MW irradiation on thermal decomposition temperatures (*T*_10_) and char yields of the PO polyimides (Table 3 and Figure S5). The *T*_10_ values of the polyimides ranged from 586 to 594 °C, and the char yields of the polyimides ranged from 83% to 87%. There were no trends in thermal properties of the control PO and MW-treated POs. Glass transition temperatures of the polyimides were not observed in differential scanning calorimetry (DSC). Collectively considering the mechanical and thermal properties of the MW-treated polyimides, the optimum MW irradiation conditions were 240 W for 2 min because the highest tensile strength and elongation at break were obtained under these conditions.

Surface and cross-section morphology of the polyimide films was investigated by field emission scanning electron microscopy (FE-SEM). The microscopic images of Control-PO and PO-240-2m are shown in Figure S6. The Control-PO and PO-240-2m films had relatively smooth and flat surfaces. 

The improvement in polyimide properties by MW treatment was next studied in the other polyimides (Table 4). MW-treated polyimide films were prepared by MW irradiation of drop-casted PAA solutions at 240 W for 2 min, followed by thermal imidization. Control polyimide films were also prepared by the conventional two-step method. The MW-treated polyimides showed higher viscosities compared to the corresponding control polyimides, leading to their higher tensile strengths than those of the corresponding control polyimides (*p* < 0.05). In particular, the MW-treated BTO film exhibited 27% greater tensile strength than the Control-BTO film (*p* < 0.05). Generally there was no significant difference between elongations at break (or modulus) values of the MW-treated samples and those of corresponding control samples (*p* > 0.05). These results indicate that the tensile strength of the polyimide films was enhanced by the MW treatment while film flexibility was maintained. MW irradiation had some effects on thermal properties, but consistent trends were not observed (Figure S7 and Table 4).

### 3.3. Model Studies

**Scheme 2:**
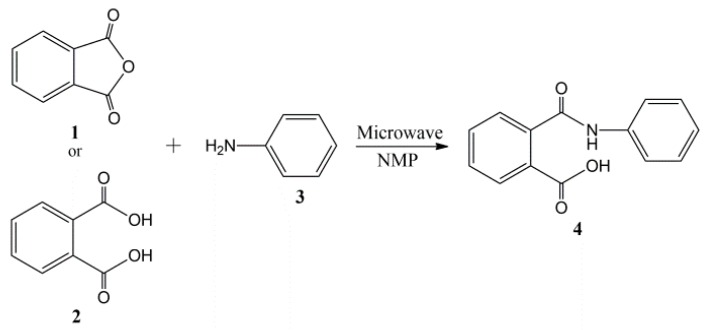
Model study of the reaction of **1** (phthalic anhydride) (or **2** (phthalic acid)) with **3** (aniline). **4**: *N*-phenylphthalamic acid.

**Figure 4:**
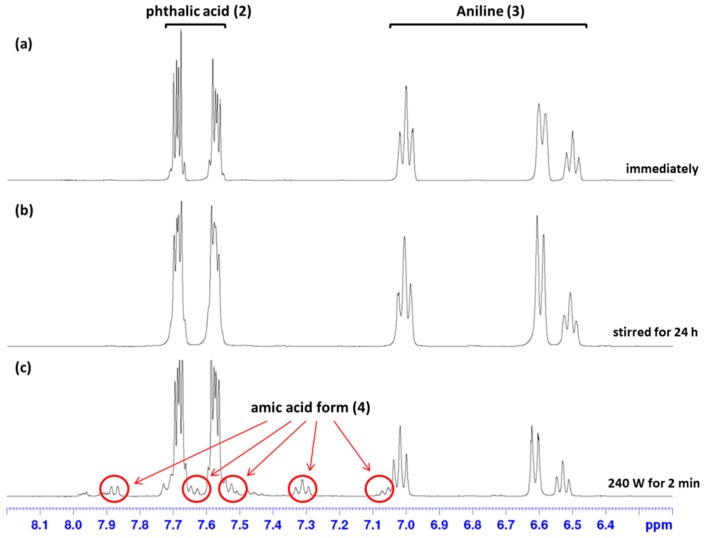
Acquired ^1^H NMR spectra of reaction mixtures of **2** and **3** in NMP (**a**) immediately after the two compounds were homogeneously mixed, (**b**) after the mixture was stirred for 24 h at room temperature, and (**c**) after the mixture was subjected to MW irradiation at 240 W for 2 min.

**Figure 5:**
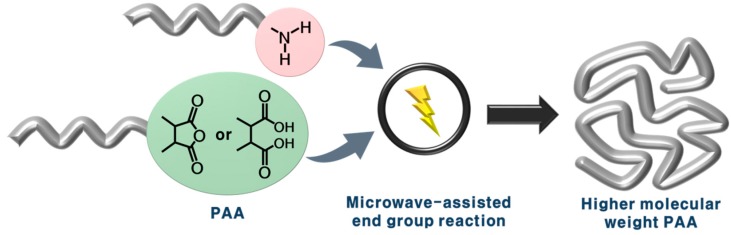
Schematic illustration of the MW-assisted end group reaction that forms higher-molecular-weight PAA.

The increases in polyimide viscosity were hypothetically attributed to the molecular weight increase due to the linking reactions between PAA end groups. We conducted model studies to verify the MW-assisted reaction between PAA end groups (Scheme 2). Experimental details are described in the Supplementary Materials. The end of the PAA chain would be in the form of anhydride, carboxyl, or amino groups. Phthalic anhydride (**1**), phthalic acid (**2**), and aniline (**3**) were chosen as reagents for the model study. When **1** and **3** were mixed in NMP at room temperature, they reacted quickly to produce *N*-phenylphthalamic acid (**4**), which was confirmed by the fact that the ^1^H NMR spectrum of the reaction product between **1** and **3** (Figure S8d) was practically identical to that of the authentic compound, **4** (Figure S8e). The reactivity of **1** and **3** was so efficient, even without MW irradiation, that the influence of MW irradiation could not be studied. On the other hand, the reaction behavior of **2** and **3** could be investigated by ^1^H NMR spectroscopy (Figure 4). No reaction was observed immediately after **2** and **3** were mixed in NMP (Figure 4a) or after stirring of the mixture for 24 h at room temperature (Figure 4b). However, when the mixture was irradiated at 240 W for 2 min, peaks of **4** appeared (Figure 4c). This result indicates that the carboxyl and amino end groups of different PAA chains react with each other under MW energy, resulting in the MW-assisted formation of higher molecular weight PAA (Figure 5).

The ability of a specific substance to convert electromagnetic energy into heat is gauged in terms of loss tangent (tan δ) [27]. NMP has a tan δ of 0.275 at a frequency of 2.45 GHz and 20 °C, which is higher than those of *N*,*N*-dimethylformamide (0.161), water (0.123), and chloroform (0.091). This indicates that NMP would absorb MW and convert it into heat efficiently, leading to a rapid increase in temperature of the reaction mixture. It was found that the reaction mixture temperature rose from room temperature to around 130 °C when the mixture was irradiated with MW at 240 W for 2 min. In addition, MW energy directly absorbed by the PAA end groups would contribute to the linking reaction [27].

It is possible that the reaction of the amino end group with the amic acid carboxyl group of different PAA chains would occur, leading to the formation of branched PAA rather than linear PAA. To investigate this possibility, a model study of the reaction of **4** with **3** was carried out. When a 1:1 molar ratio mixture of **4** and **3** in NMP was irradiated with MW at 240 W for 2 min, only a small amount of *N*-phenylphthalimide (**5**) formation was observed (Scheme S1 and Figure S9). This result rules out the possibility of the MW-assisted reaction of amino end group with amic acid carboxyl groups of PAA, producing branched PAA. Based on the experimental results, including the model studies, we found that MW irradiation of PAA solutions specifically induced end-group linking reactions and partial imidization of PAAs.

## 4. Conclusions

Polyimide films were prepared by MW irradiation of the drop-casted PAA solutions and subsequent thermal imidization. The MW-treated polyimide films exhibited up to 27% greater tensile strength and up to 32% greater elongation at break than the control films. Generally tensile strength was increased while film flexibility was maintained or increased. Considering the mechanical properties, MW power, and irradiation time, the optimum MW irradiation conditions were determined to be 240 W for 2 min. The increase in the mechanical properties can be attributed to the increase in polyimide molecular weight through the reaction between carboxyl and amino end groups of different PAA chains by MW irradiation. This hypothesis was supported by model studies in which the reaction of phthalic acid with aniline occurred to produce **4** by MW irradiation. Thus, polyimide films with improved mechanical properties can be simply and easily prepared by MW irradiation. The process requires relatively low MW power, a very short amount of time, and no inorganic reinforcement.

## Figures and Tables

**Table 1:** MW irradiation conditions and inherent viscosities of PO polyimides.

Polyimide ^a^	MW Power (W)	MW Irradiation Time (min)	Inherent Viscosity (dL/g)
Control-PO ^b^	-	-	1.23
PO-80-120m	80	120	1.64
PO-240-2m	240	2	1.68
PO-400-1m	400	1	1.36

^a^ As a representative example, PO-240-2m is a polyimide film prepared from PMDA and ODA by MW irradiation of a drop-casted PAA-PO solution at 240 W for 2 min and subsequent thermal imidization. ^b^ Control-PO is a polyimide film prepared from PMDA and ODA by the conventional two-step method without MW irradiation.

**Table 2:** Degree of imidization (DI) of the PAA-POs as measured by FT-IR spectroscopy.

PAA ^a^	Degree of Imidization (%) ^b^	A_1377_^c^	A_1500_^c^	A_1377_/A_1500_
PAA-PO-80-120m	11	0.013481	0.235130	0.057334
PAA-PO-240-2m	10	0.001469	0.027425	0.053564
PAA-PO-240-3m	50	0.113597	0.415148	0.273630
PAA-PO-400-1m	6	0.002146	0.067276	0.031898
PAA-PO-400-2m	14	0.007818	0.104859	0.074557
PAA-PO-640-1m	20	0.013738	0.125557	0.109416

^a^ As a representative example, PAA-PO-240-2m is a PAA-PO powder obtained by MW irradiation of a drop-casted PAA-PO solution at 240 W for 2 min, followed by reprecipitation. ^b^ Determined using Equation (1) (see Section 2.6). ^c^ A_1377_ and A_1500_ are the absorbance values of the 1377 and 1500 cm^−1^ absorption bands of PAA-PO, respectively.

**Table 3:** Mechanical and thermal properties of the PO polyimides ^a^.

Polyimide ^b^	Tensile Strength (MPa)	Elongation at Break (%)	Tensile Modulus (GPa)	*T*_10_ (°C) ^d^	Char Yield (%) ^e^
Control-PO ^c^	118.2 (2.3) *	50.5 (2.7) ^†^	2.7 (0.7) ^‡^	588	84
PO-80-120m	130.2 (3.6) **	60.6 (5.1) ^††^	2.3 (0.4) ^‡^	586	83
PO-240-2m	144.1 (6.5) ***	66.8 (3.0) ^††^	2.5 (0.5) ^‡^	590	84
PO-400-1m	120.3 (5.3) *	51.1 (6.1) ^†^	1.8 (0.4) ^‡^	594	87

^a^ Mechanical property data was analyzed by statistical analysis. Parentheses show standard deviation of five replicates. Values with the same symbol are not significantly different (*p* > 0.05). ^b^ As a representative example, PO-240-2m is a polyimide film prepared from PMDA and ODA by MW irradiation of a drop-casted PAA-PO solution at 240 W for 2 min and subsequent thermal imidization. ^c^ Control-PO is a polyimide film prepared by drop-casting of a PAA-PO solution and subsequent thermal imidization without MW irradiation. ^d^ The temperature at which a specimen shows 10 wt % decomposition under nitrogen. ^e^ Char yield at 600 °C under nitrogen.

**Table 4:** Viscosities and mechanical and thermal properties of the polyimides ^a^.

Polyimide ^b^	Inherent Viscosity (dL/g) ^c^	Tensile Strength (MPa)	Elongation at Break (%)	Tensile Modulus (GPa)	*T*_10_ (°C) ^d^	Char Yield (%) ^e^
Control-BPO	1.81	139.1 (4.4) ^$^	55.2 (3.0) *	3.0 (0.9) ^†^	597	89
BPO-240-2m	2.21	147.8 (4.1) ^$$^	59.1 (5.2) *	2.7 (0.6) ^†^	590	87
Control-BTO	-	98.1 (2.1) ^#^	5.6 (0.9) **	5.7 (0.5) ^††^	582	85
BTO-240-2m	-	124.6 (5.4) ^##^	5.7 (1.6) **	2.7 (0.3) ^†††^	571	81
Control-6FO	1.06	110.0 (3.5) ^&^	7.4 (1.4) ***	3.4 (0.3) ^††††^	549	71
6FO-240-2m	1.21	121.1 (4.6) ^&&^	8.1 (0.6) ***	3.2 (0.3) ^††††^	550	72
Control-HPO	1.13	71.6 (1.6) ^^^	5.0 (1.2) ****	2.9 (0.8) ^†††††^	497	44
HPO-240-2m	1.21	75.7 (5.4) ^^^^	5.4 (1.1) ****	2.6 (0.6) ^†††††^	499	62

^a^ Mechanical property data was analyzed by statistical analysis. Parentheses show standard deviation of five replicates. Values with the same symbol are not significantly different (*p* > 0.05). ^b^ BPO: BPDA-ODA; BTO: BTDA-ODA; 6FO: 6FDA-ODA; HPO: HPMDA-ODA. As a representative example, BPO-240-2m is a polyimide film prepared from BPDA and ODA by MW irradiation of a drop-casted PAA-BPO solution at 240 W for 2 min and subsequent thermal imidization. ^c^ Control-BTO and BTO-240-2m were not insoluble in concentrated sulfuric acid. ^d^ The temperature at which a specimen shows 10 wt % decomposition under nitrogen. ^e^ Char yield at 600 °C under nitrogen.

## Supplementary Materials

The following are available online at https://www.mdpi.com/2073-4360/11/3/477/s1, Figure S1: FT-IR-spectra of polyimides: (**a**) BPO-240-2m, (**b**) BTO-240-2m, (**c**) 6FO-240-2m, and (**d**) HPO-240-2m; Figure S2: FT-IR spectra of PAA-POs: (**a**) PAA-PO-240-3m, (**b**) PAA-PO-400-2m, and (**c**) PAA-PO-640-1m; Figure S3. FT-IR spectra of PAA-POs and a reference PO polyimide; Figure S4: Stress–strain curves of PO films; Figure S5: TGA curves of PO polyimides; Figure S6: SEM images: Surface of (**a**) Control-PO and (**b**) PO-240-2m films; cross-section of (**c**) Control-PO and (**d**) PO-240-2m films; Figure S7: TGA curves of the polyimides; Figure S8: ^1^H NMR spectra of (**a**) phthalic anhydride (**1**), (**b**) phthalic acid (**2**), (**c**) aniline (**3**), (**d**) the isolated product from the reaction of **1** with **3** in NMP for less than 1 min, and (**e**) an authentic *N*-phenylphthalamic acid (**4**); Figure S9: ^1^H NMR spectra of (**a**) the isolated product from the reaction of **4** with **3** in NMP by MW irradiation at 240 W for 2 min, (**b**) the crude product from the reaction of **4** with **3** in NMP by MW irradiation at 240 W for 2 min, and (**c**) an authentic N-phenylphthalimide (**5**); Scheme S1: Model study of the reaction of **4** with **3**; Table S1: Elemental analysis of the polyimides.
