# Supplementary material for: Enhancement of the Mechanical Properties of Polyimide Film by Microwave Irradiation
Source: Polymers (Basel). 2019 Mar 12;11(3):477. doi: 10.3390/polym11030477 (PMC6473371; doi:10.3390/polym11030477)
Supplement: Supplementary file 1 [file polymers-11-00477-s001.pdf]

<Supplementary Materials>

# Enhancement of the mechanical properties of polyimide film by microwave irradiation

Ju-Young Choi, Seung-Won Jin, Dong-Min Kim, In-Ho Song, Nam-Kyeong Nam, Hyeong-Joo Park

and Chan-Moon Chung*

Department of Chemistry, Yonsei University, Wonju, Gangwon-do 26493, Republic of Korea

*Correspondence to: Chan-Moon Chung (E-mail: cmchung@yonsei.ac.kr)

## Model Study

*Model study of reaction of phthalic anhydride (**1**) with aniline (**3**)*

A dried 50-mL round-bottom flask was charged with **1** (1.481 g, 0.0100 mol) and **3** (0.9313 g, 0.0100 mol) in NMP (21.1 mL) under nitrogen atmosphere. After **1** was dissolved, **3** was added, and within 1 min the mixture was poured into distilled water, forming a precipitate that was collected by filtration. Washing with water followed by drying in vacuum afforded a white powder (1.734 g, 72% yield). The $^{1}H$ NMR spectrum of the product (Figure S4d) is practically identical to that of an authentic compound of *N*-phenylphthalamic acid (**4**) (Figure S4e).

*Model study of reaction of phthalic acid (**2**) with **3***

A dried 50-mL round-bottom flask was charged with **2** (1.661 g, 0.0100 mol) and **3** (0.9313 g, 0.0100 mol) in NMP (22.7 mL) under nitrogen atmosphere. $^{1}H$ NMR spectroscopy was performed using reaction mixture samples after the following three experiments: (1) immediately after **2** and **3** were homogeneously mixed (the reaction time was within 1 min) (Figure 3a), (2) after the mixture solution was stirred at room temperature for 24 h (Figure 3b), and (3) after the mixture solution was drop-cast onto slide glass and then irradiated with MW at 240 W for 2 min (Figure 3c).

*Model study of reaction of N-phenylphthalamic acid (**4**) with **3***

In a dried 20-mL vial, **4** (0.241 g, 0.00100 mol) and **3** (0.0931 g, 0.00100 mol) were dissolved in NMP (2.9 mL). The solution was drop-cast onto slide glass and then irradiated with MW at 240 W for 2 min. The resultant solution was poured into distilled water, forming a precipitate that was collected by filtration. Washing with water followed by drying in vacuum afforded a white powder. The product was analysed by $^{1}H$ NMR spectroscopy (Figure S5a).

*Preparation of authentic **4***

A dried 50-mL round-bottom flask was charged with **1** (1.481 g, 0.0100 mol) and **3** (0.9313 g, 0.0100 mol) in NMP (21.1 mL) under nitrogen atmosphere. This mixture was stirred for 24 h at room temperature. A white solid was collected by filtration and dried in vacuum at 50 °C. FT-IR $\nu_{max}$ (KBr): 1722, 1656, 1549 $cm^{-1}$. $^{1}H$ NMR (DMSO-$d_6$, 400 MHz, Figure S4e): δ 13.01 (s, 1H), 10.32 (s, 1H), 7.87–7.89 (d, 1H), 7.65–7.71 (m, 3H), 7.54–7.59 (m, 2H), 7.31–7.35 (m, 2H), 7.05–7.09 ppm (m, 1H). $^{13}C$ NMR (DMSO-$d_6$, 100 MHz): δ 168.0, 167.8, 140.0, 139.3, 132.2, 130.4, 130.0, 129.9, 129.1, 128.3, 123.8, 119.9 ppm.

*Preparation of authentic N-phenylphthalimide (**5**)*

A 25-mL vial was charged with **1** (0.741 g, 0.0050 mol) and **3** (0.466 g, 0.0050 mol) in NMP (10.5 mL). The mixture was irradiated with MW at 240 W for 10 min. The reaction mixture was poured into distilled water, forming a precipitate that was collected by filtration. Washing with water followed by drying in vacuum afforded a white powder. FT-IR $\nu_{max}$ (KBr): 1779, 1709, 1386 cm$^{-1}$. $^{1}$H NMR (DMSO-$d_6$, 400 MHz, Figure S5b): $\delta$ 7.92–7.96 (m, 2H), 7.89–7.91 (m, 2H), 7.52–7.55 (m, 2H), 7.43–7.46 ppm (m, 3H). $^{13}$C NMR (DMSO- $d_6$, 100 MHz): $\delta$ 167.5, 135.2, 132.4, 132.0, 129.3, 128.6, 127.9, 123.9 ppm.

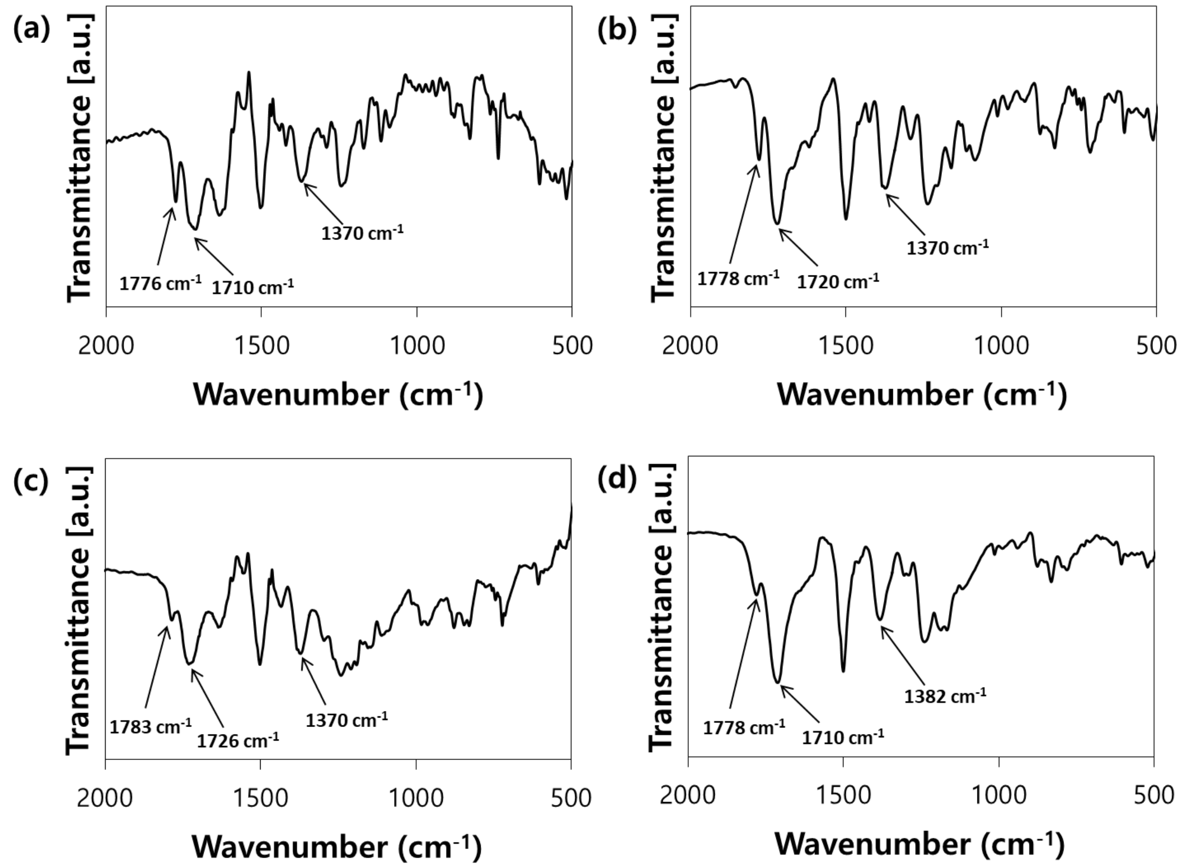


**Figure S1.** FT-IR-spectra of polyimides: (a) BPO-240-2m, (b) BTO-240-2m, (c) 6FO-240-2m and (d) HPO-240-2m.

FT-IR (KBr, cm$^{-1}$): (a) 1776 (imide C=O asymmetric stretch), 1710 (imide C=O symmetric stretch), 1370 (imide C–N stretch), (b) 1778 (imide C=O asymmetric stretch), 1720 (imide C=O symmetric stretch), 1370 (imide C–N stretch), (c) 1783 (imide C=O asymmetric stretch), 1726 (imide C=O symmetric stretch), 1370 (imide C–N stretch), (d) 1778 (imide C=O asymmetric stretch), 1710 (imide C=O symmetric stretch), 1382 (imide C–N stretch).

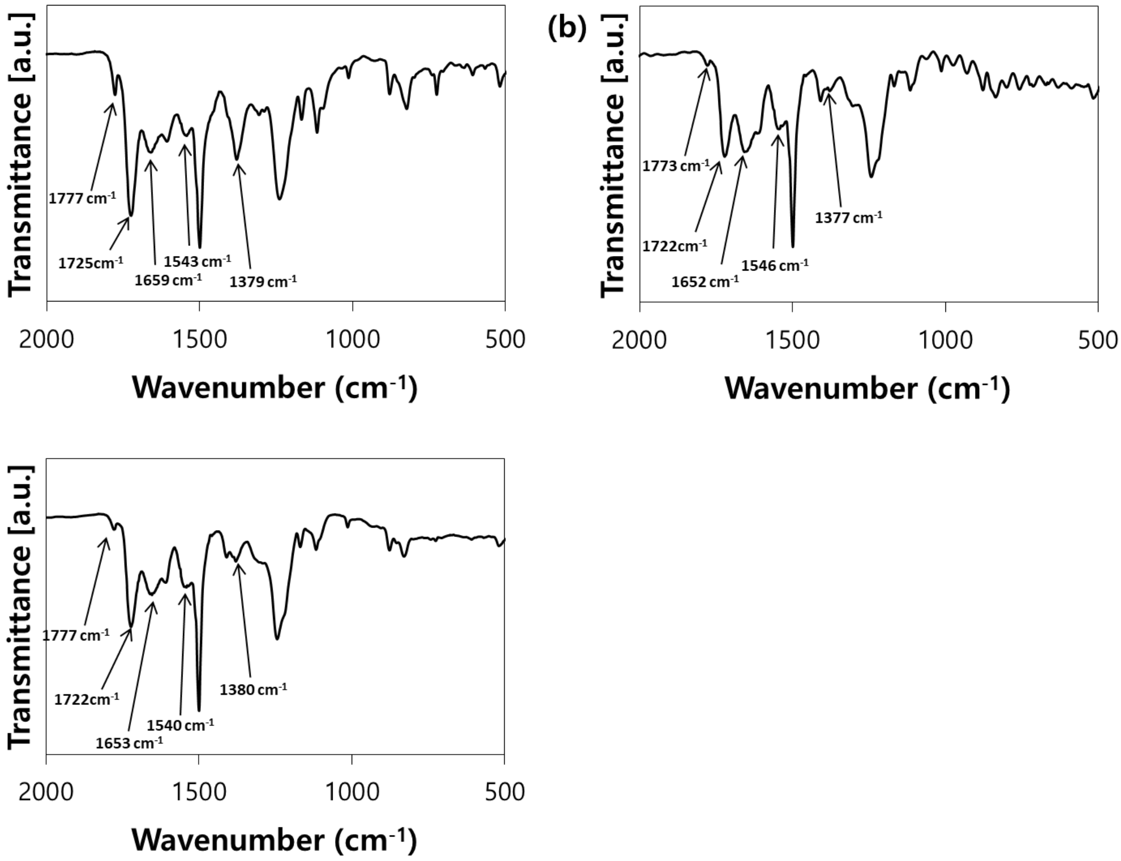


**Figure S2.** FT-IR-spectra of PAA-POs: (a) PAA-PO-240-3m, (b) PAA-PO-400-2m and (c) PAA-PO-640-1m.

FT-IR (KBr, $cm^{-1}$): (a) 1777 (imide C=O asymmetric stretch), 1725 (carboxyl C=O stretch), 1659 (amide C=O stretch), 1543 (amide C-N stretch), 1379 (imide C–N stretch), (b) 1773 (imide C=O asymmetric stretch), 1722 (carboxyl C=O stretch), 1652 (amide C=O stretch), 1546 (amide C-N stretch), 1377 (imide C–N stretch), (c) 1777 (imide C=O asymmetric stretch), 1722 (carboxyl C=O stretch), 1653 (amide C=O stretch), 1540 (amide C-N stretch), 1380 (imide C–N stretch).

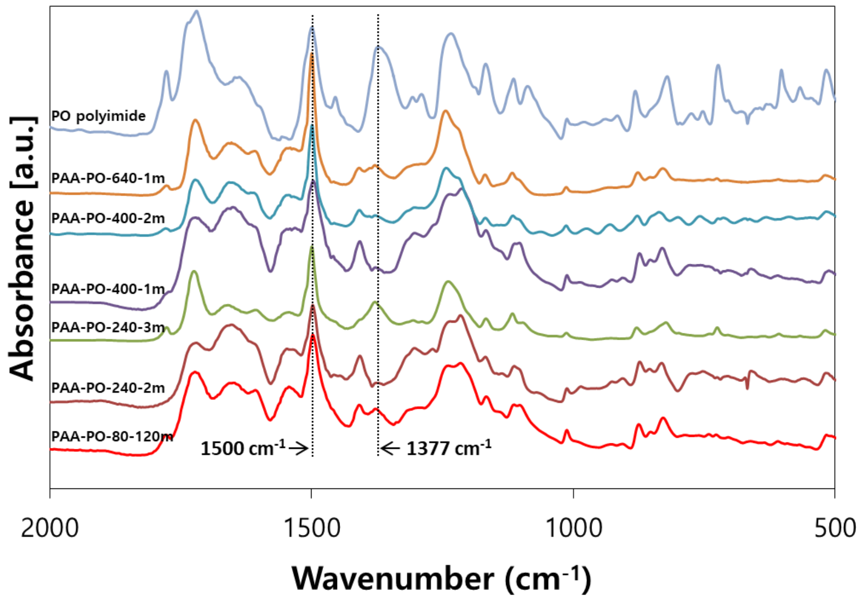


**Figure S3.** FT-IR spectra of PAA-POs and a reference PO polyimide.

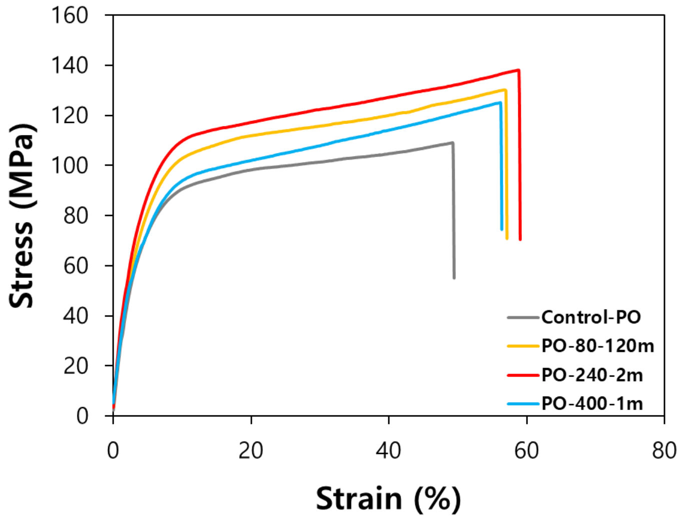


**Figure S4.** Stress-strain curves of PO films.

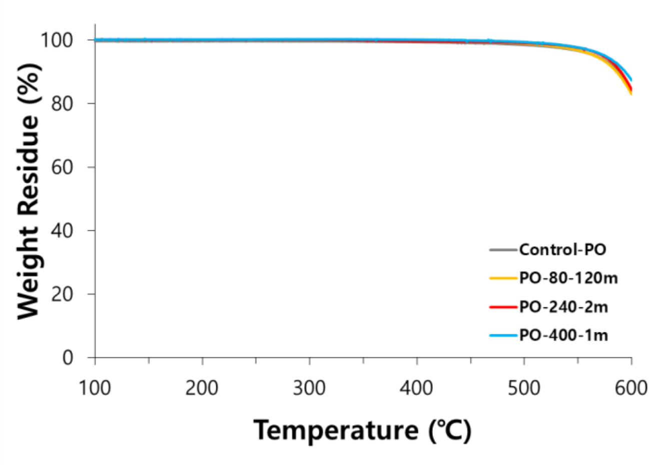


**Figure S5.** TGA curves of PO polyimides.

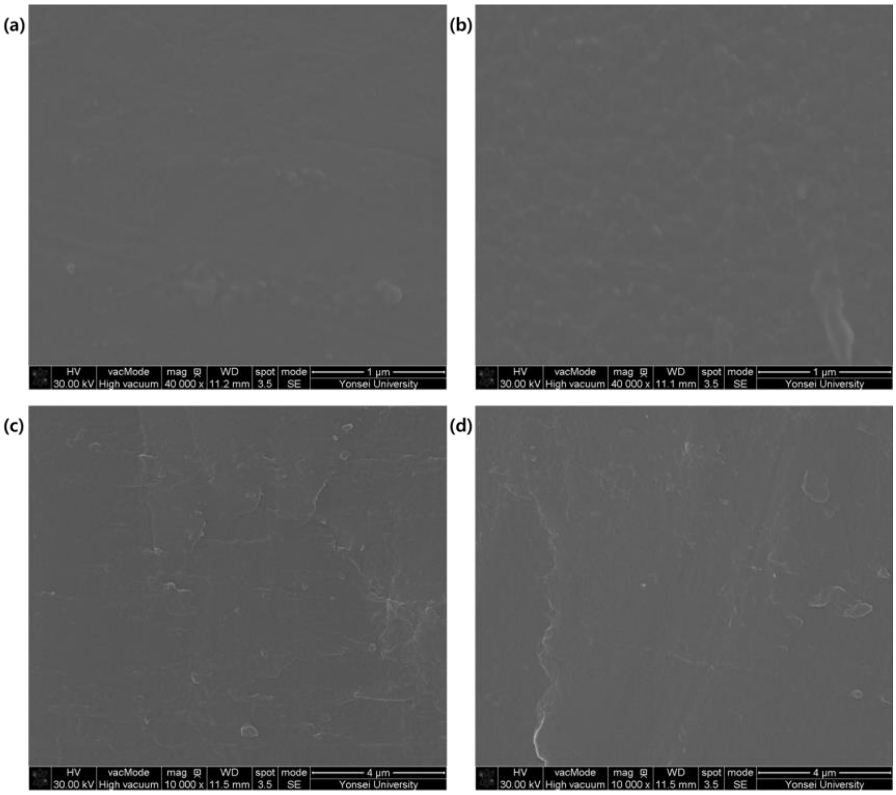


**Figure S6**. SEM images: surface of (a) Control-PO and (b) PO-240-2m films; cross section of (c) Control-PO and (d) PO-240-2m films.

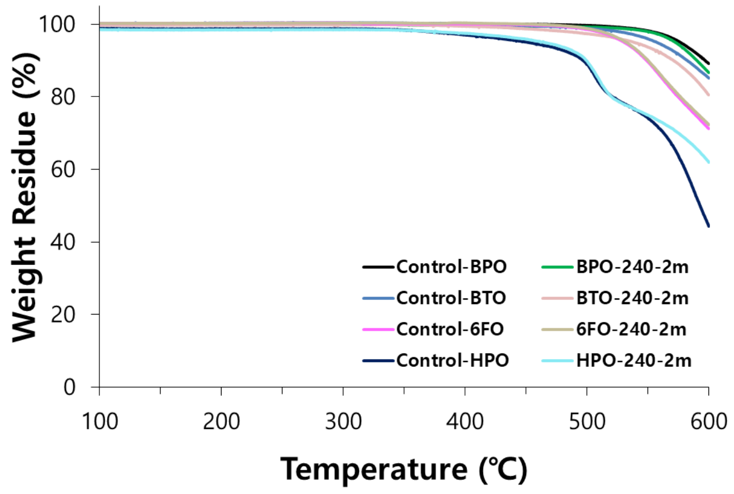


**Figure S7.** TGA curves of the polyimides.

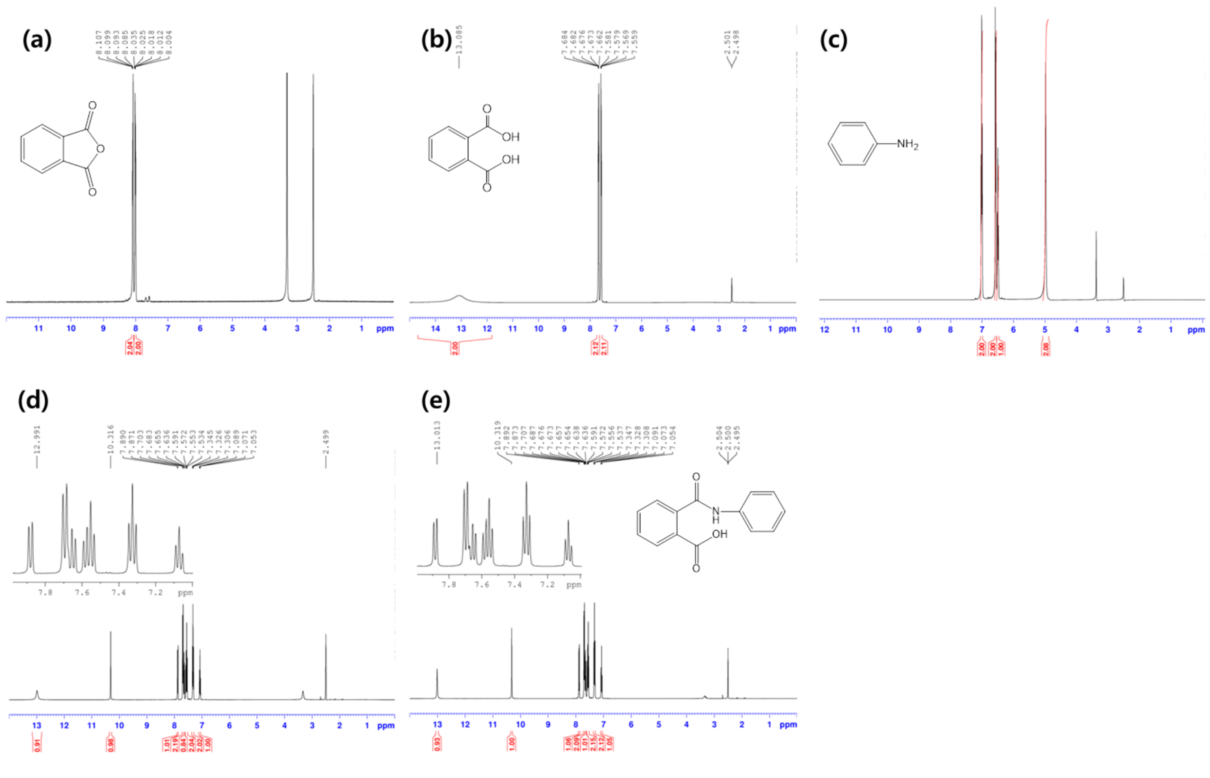


**Figure S8.** $^{1}$H NMR spectra of (a) phthalic anhydride (**1**), (b) phthalic acid (**2**), (c) aniline (**3**), (d) the isolated product from the reaction of **1** with **3** in NMP for less than 1 min and (e) an authentic *N*-phenylphthalamic acid (**4**).

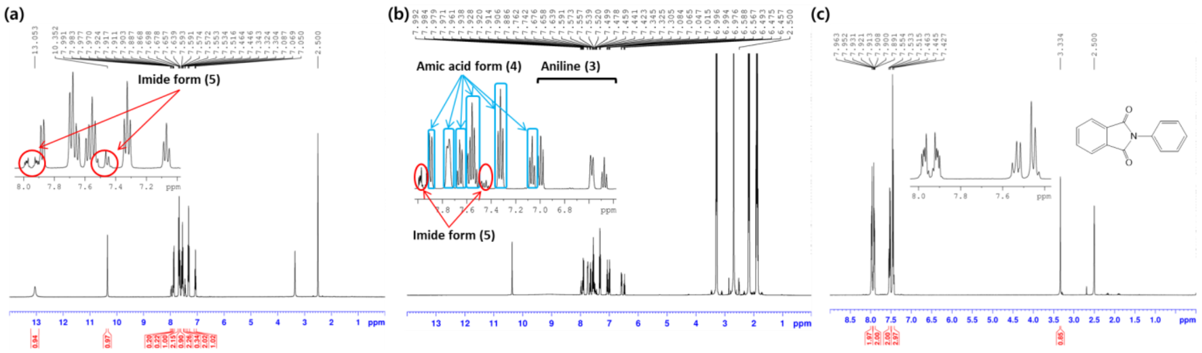


**Figure S9.** $^{1}$H NMR spectra of (a) the isolated product from the reaction of **4** with **3** in NMP by MW irradiation at 240 W for 2 min, (b) the crude product from the reaction of **4** with **3** in NMP by MW irradiation at 240 W for 2 min and (c) an authentic *N*-phenylphthalimide (**5**).

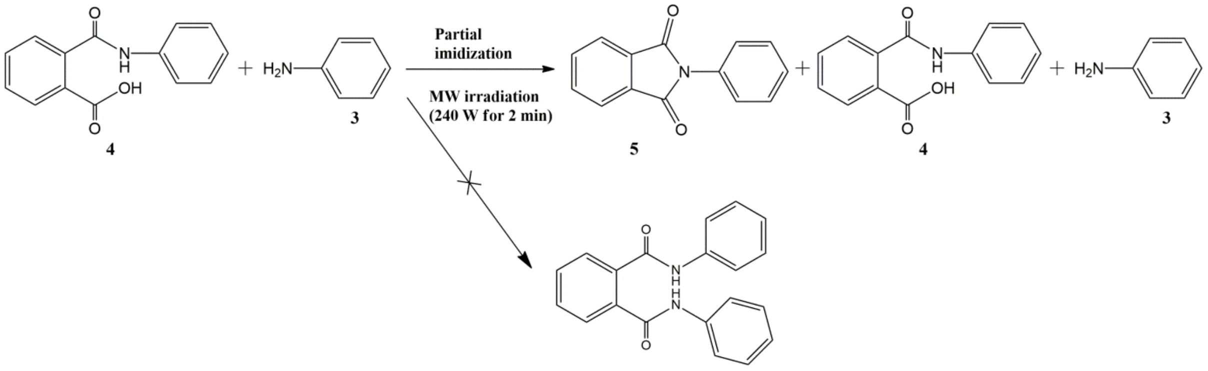


**Scheme S1.** Model study of the reaction of **4** with **3**.

**Table S1.** Elemental analysis of the polyimides.

| Polyimide[a,b] | | Elemental Analysis (%) | | |
|---|---|---|---|---|
| | | **C** | **H** | **N** |
| Control-PO | Calcd. | 69.11 | 2.63 | 7.33 |
| | Found | 67.49 | 2.63 | 7.27 |
| PO-240-2m | Calcd. | 69.11 | 2.63 | 7.33 |
| | Found | 67.66 | 2.74 | 7.44 |
| Control-BPO | Calcd. | 73.36 | 3.08 | 6.11 |
| | Found | 72.59 | 3.10 | 6.18 |
| BPO-240-2m | Calcd. | 73.36 | 3.08 | 6.11 |
| | Found | 72.50 | 3.12 | 6.23 |
| Control-BTO | Calcd. | 71.61 | 2.90 | 5.76 |
| | Found | 70.56 | 2.97 | 6.52 |
| BTO-240-2m | Calcd. | 71.61 | 2.90 | 5.76 |
| | Found | 70.83 | 3.02 | 6.60 |
| Control-6FO | Calcd. | 61.19 | 2.32 | 4.60 |
| | Found | 60.53 | 2.36 | 4.68 |
| 6FO-240-2m | Calcd. | 61.19 | 2.32 | 4.60 |
| | Found | 60.32 | 2.47 | 4.71 |
| Control-HPO | Calcd. | 68.04 | 4.15 | 7.21 |
| | Found | 64.90 | 4.15 | 7.25 |
| HPO-240-2m | Calcd. | 68.04 | 4.15 | 7.21 |
| | Found | 64.62 | 4.22 | 7.01 |

[a] PO: PMDA/ODA; BPO: BPDA/ODA; BTO: BTDA/ODA; 6FO: 6FDA/ODA; HPO: HPMDA/ODA. As a representative example, BPO-240-2m is a polyimide film prepared from BPDA and ODA by MW irradiation of a drop-casted PAA-BPO solution at 240 W for 2 min and subsequent thermal imidization. [b] Control polyimides are polyimide films were prepared by the conventional two-step method without MW irradiation.
